# Supplementary material for: Exploring the Functional Heterogeneity of Directly Reprogrammed Neural Stem Cell-Derived Neurons via Single-Cell RNA Sequencing
Source: Cells. 2023 Dec 11;12(24):2818. doi: 10.3390/cells12242818 (PMC10742074; doi:10.3390/cells12242818)
Supplement: Supplementary file 1 [file cells-12-02818-s001.zip › cells-2711164-supplementary.pdf]

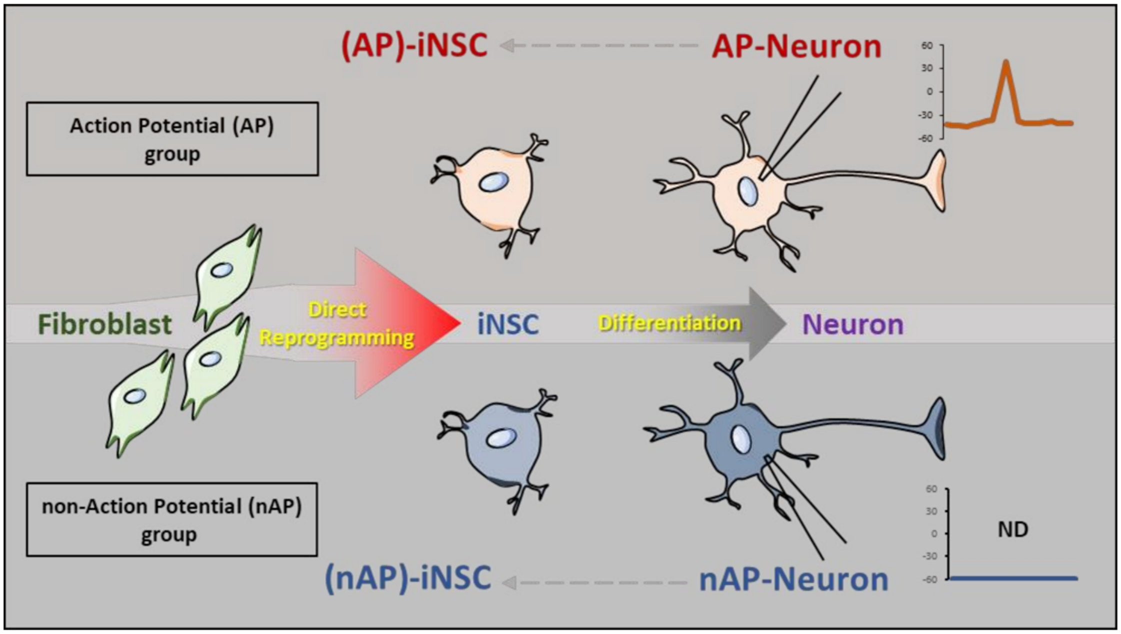

Figure S1. Experimental scheme related to Figures 1 to 6. This diagram shows the process to generate induced neural stem cells (iNSCs) from fibroblasts through direct reprogramming. Neurons derived from the iNSCs were evaluated for electrophysiological function using patch-clamp analysis. Our samples were divided into four groups based on the presence or absence of action potential (AP) in the neurons. The AP Neuron group exhibited an action potential, which differentiated them from (AP)-iNSCs. In the nAP Neuron group, derived from (nAP)-iNSCs, no action potential was detected.

A

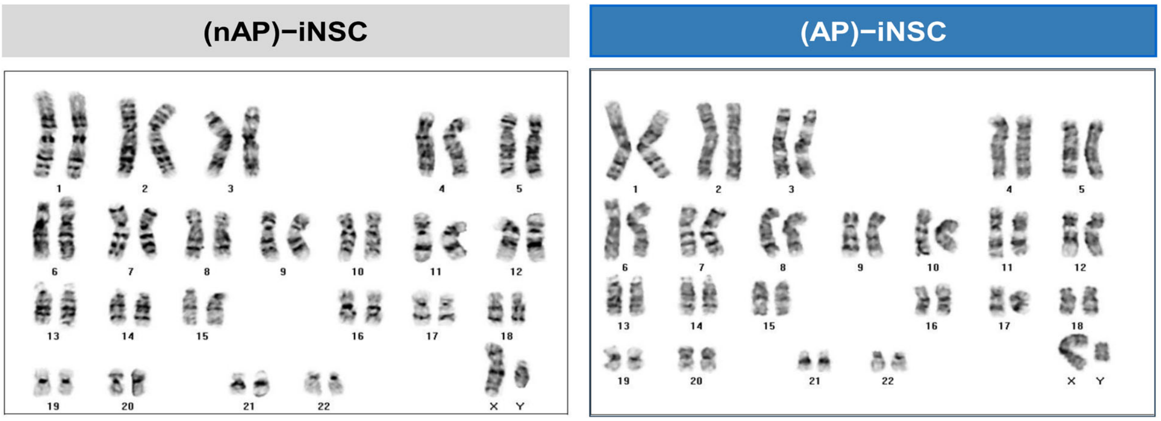

B

	Amelogenin	D13S317	D16S539	CSF1PO	TH01	vWA	D7S820	D58S818	TPOX
**hFB**	XY	11, 12	9, 11	12, 13	6, 9.3	17, 18	12	11, 12	10, 11
**(nAP) −iNSC**	XY	11, 12	9, 11	12, 13	6, 9.3	17, 18	12	11, 12	10, 11
**(AP) −iNSC**	XY	11, 12	9, 11	12, 13	6, 9.3	17, 18	12	11, 12	10, 11

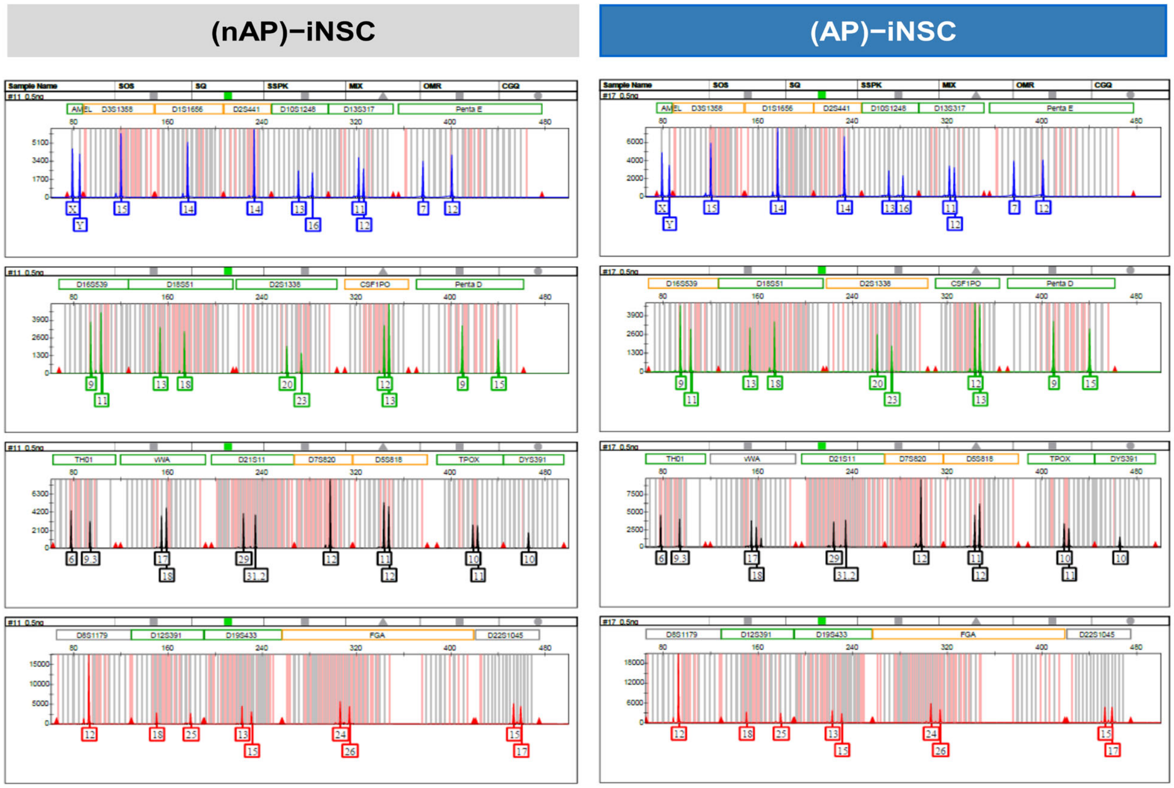

Figure S2. Genetic stability test for induced neural stem cells (iNSCs). (A) Karyotyping analysis of directly reprogrammed iNSCs. Both (nAP)-iNSC and (AP)-iNSCs revealed stable and normal chromosome morphologies compared to human fibroblasts. (B) Both short tandem repeat (STR) profiles of (nAP)-iNSCs and (AP)-iNSCs were matched with the cell source of human fibroblasts for nine STR loci (CRL2097, ATCC) (upper table). Also shown are representative electropherograms of 24 STR loci for (nAP)-iNSCs and (AP)-iNSCs (lower panel). hFB, human fibroblast; (nAP)-iNSC, iNSC group, which is the source cell of nAP (non-action potential) neurons; (AP)-iNSC, iNSC group, which is the source cell of AP Neurons.

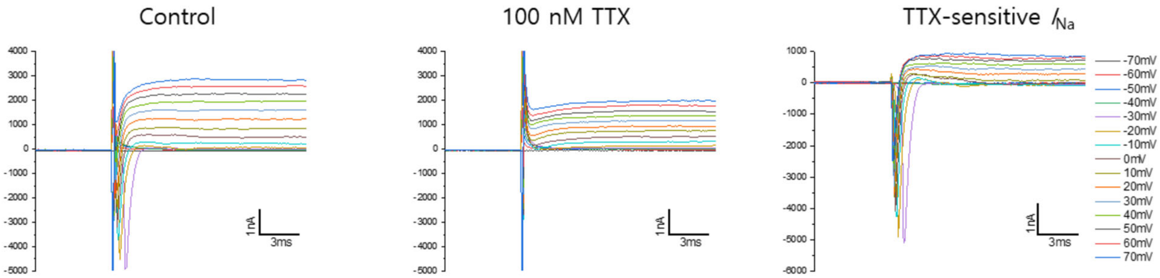

Figure S3. Representative images of the electrophysiological properties of induced neural stem cell (iNSC)-derived AP Neurons from whole-cell patch-clamp recordings. Tetrodotoxin-sensitive introverted Na+ currents in the AP Neuron group under different intensities of electrical stimulation (0–70 mV). The iNSCs did not show any action potential.

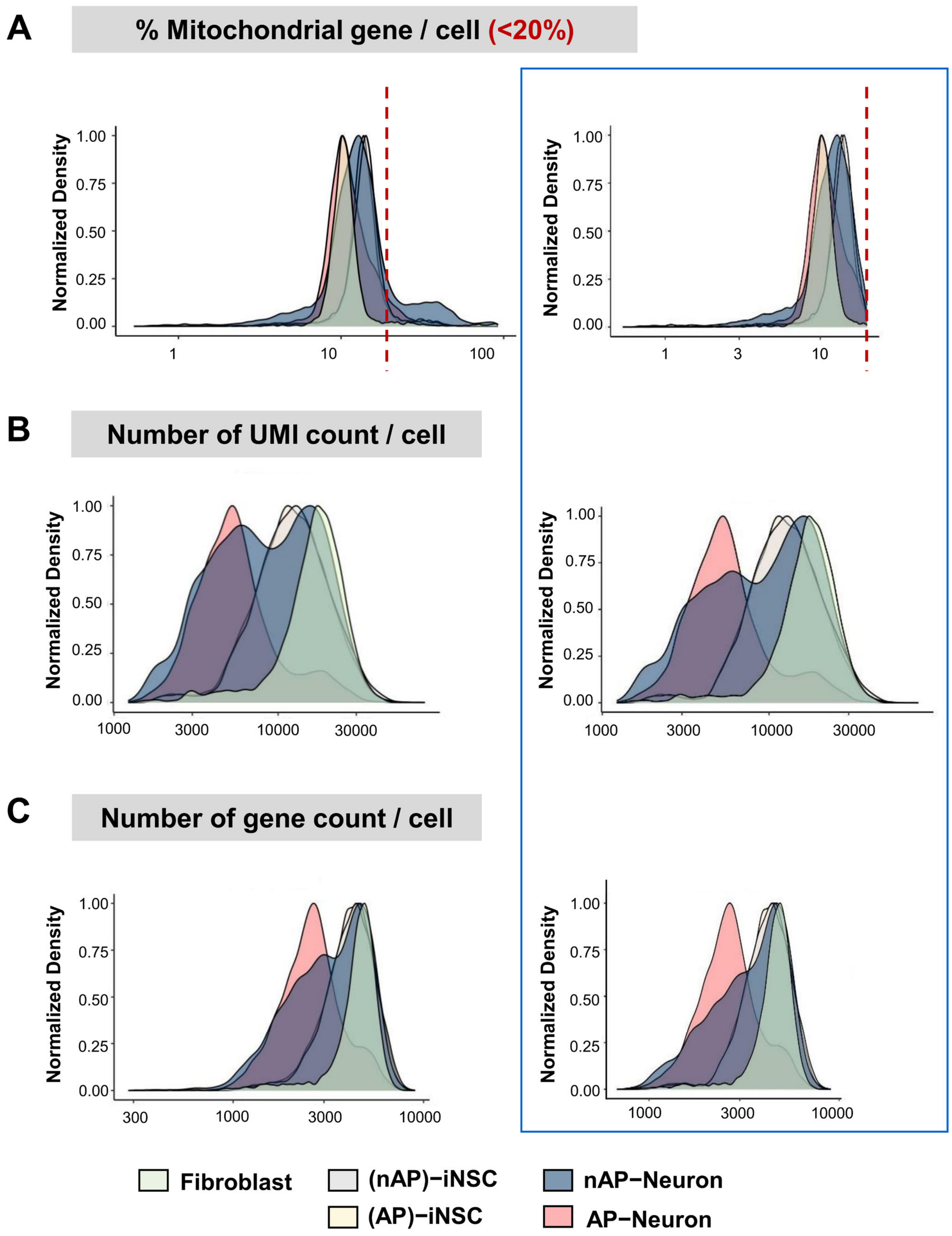

Figure S4. Quality criteria used for clustering and analysis. Quality control data for each cell type of sample were verified by mitochondrial RNA percentage, UMI (unique molecular identifier) counts, and gene counts. (A) Cells expressing over 20% of mitochondrial genes were excluded from further clustering analysis. (B,C) The distribution of the UMI and gene counts for each group.

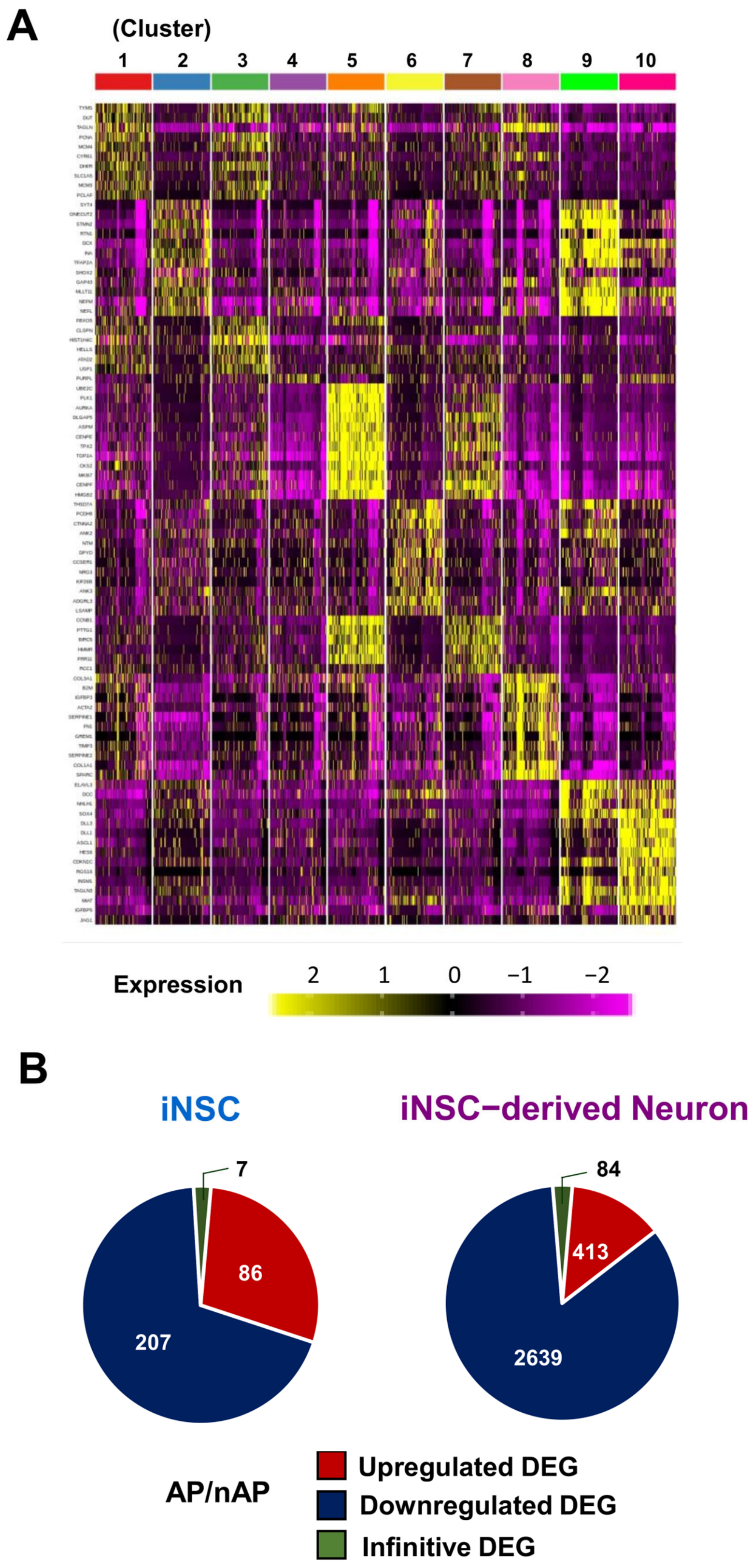

Figure S5. Global gene expression changes between the AP and nAP groups. (A) Heatmap of top-ranked gene expressions in each cluster. (B) Differentially expressed genes (DEG) in the AP group samples compared to each nAP group sample. Infinitive DEG means at least one sample did not have certain gene expression.

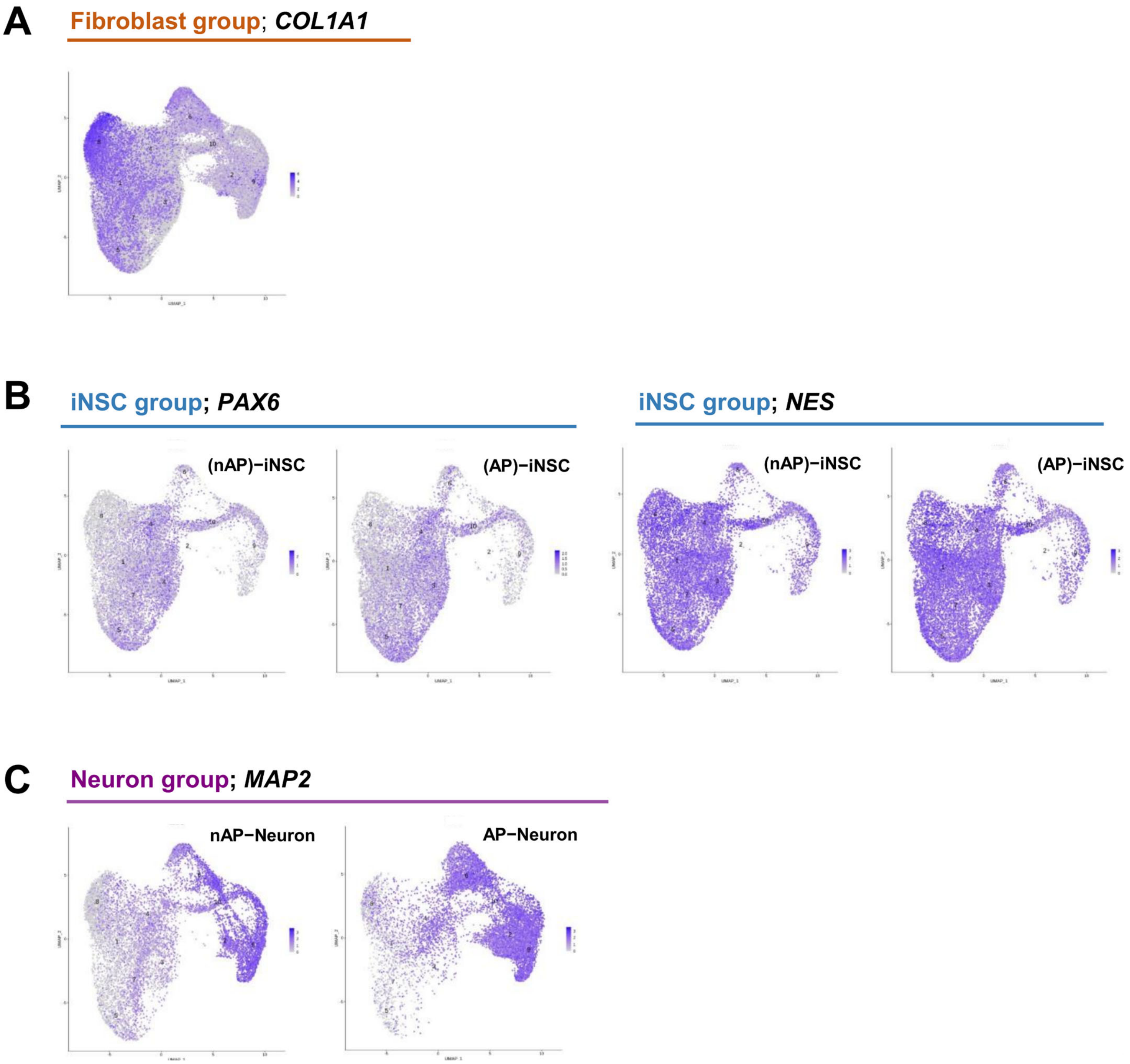

Figure S6. Cell type marker expression changes. Samples were feature-plotted with the appropriate marker expressed: (A) fibroblast: COL1A1, (B) iNSCs: PAX6 and NES, (C) neurons: MAP2. Table S1: primer sequences used to characterize the cells used in this study.
